# Study on the Antimicrobial Properties of Citrate-Based Biodegradable Polymers

**DOI:** 10.3389/fbioe.2014.00023

**Published:** 2014-07-03

**Authors:** Lee-Chun Su, Zhiwei Xie, Yi Zhang, Kytai Truong Nguyen, Jian Yang

**Affiliations:** ^1^Department of Bioengineering, University of Texas at Arlington, Arlington, TX, USA; ^2^Department of Biomedical Engineering, Materials Research Institute, The Huck Institutes of The Life Sciences, The Pennsylvania State University, University Park, PA, USA

**Keywords:** antimicrobial polymers, citric acid, crosslinking, biodegradable elastomers, wound dressing

## Abstract

Citrate-based polymers possess unique advantages for various biomedical applications since citric acid is a natural metabolism product, which is biocompatible and antimicrobial. In polymer synthesis, citric acid also provides multiple functional groups to control the crosslinking of polymers and active binding sites for further conjugation of biomolecules. Our group recently developed a number of citrate-based polymers for various biomedical applications by taking advantage of their controllable chemical, mechanical, and biological characteristics. In this study, various citric acid derived biodegradable polymers were synthesized and investigated for their physicochemical and antimicrobial properties. Results indicate that citric acid derived polymers reduced bacterial proliferation to different degrees based on their chemical composition. Among the studied polymers, poly(octamethylene citrate) showed ~70–80% suppression to microbe proliferation, owing to its relatively higher ratio of citric acid contents. Crosslinked urethane-doped polyester elastomers and biodegradable photoluminescent polymers also exhibited significant bacteria reduction of ~20 and ~50% for *Staphylococcus aureus* and *Escherichia coli*, respectively. Thus, the intrinsic antibacterial properties in citrate-based polymers enable them to inhibit bacteria growth without incorporation of antibiotics, silver nanoparticles, and other traditional bacteria-killing agents suggesting that the citrate-based polymers are unique beneficial materials for wound dressing, tissue engineering, and other potential medical applications where antimicrobial property is desired.

## Introduction

Biodegradable polymeric materials play a major role in medical and pharmaceutical domains because various biomedical devices/prostheses made with these materials have contributed enormously in human health such as tissue engineering scaffolds (Flanagan and Pandit, 2003), drug delivery systems, and wound dressings (Claudia Valenta, 2003). However, a universal challenge of applying these devices *in vivo* is the infection occurred by microbial-contamination. Serious infection complications include tissue destruction, premature device failure, and the spread of the infection to other areas (Woo et al., 2000). In addition, the proliferation of microorganism stimulates the cascade of body defensive responses that can be life-threatening (Christensen et al., 1990). Bacterial infection is also a major obstacle for wound healing, especially chronic wound healing (Martin, 1997). Generally, biomaterials used in Food and Drug Administration (FDA) approved wound dressings and other implants include naturally derived materials (e.g., collagen and alginate) and synthetic polymers [e.g., polylactic acid (PLA), and poly(lactic-co-glycolic acid) (PLGA)]. However, these commonly used biomaterials do not possess intrinsic antibacterial properties. In recent years, tremendous efforts have been made to prevent and control biomaterials or implant related infections. However, traditional methods mostly rely on the incorporation of antimicrobial drugs/nanoparticles/peptides into the device matrix or a coating of antibiotics on the device surface with limited effectiveness (Leipziger et al., 1985; Costache et al., 2010; Wiegand et al., 2011). The main challenge is the rapid loss of antibiotics and the compromise of device or material functionalities including mechanical properties, degradation rate, and biocompatibility (Bach et al., 1994; O’Meara et al., 2000).

Recently, a series of citrate-based biodegradable polymers for tissue engineering, drug delivery, wound dressing, and bioimaging applications have been developed in our lab (Yang et al., 2004, 2009; Dey et al., 2008; Gyawali et al., 2010a; Tran et al., 2010). The essential component of these materials, citric acid (CA), is naturally contained in the body as an important intermediate in the metabolism, particularly, the tricarboxylic acid (TCA) cycle. It is also widely used as additives in foods/drinks and fillers in dental treatments (Scelza et al., 2004). In addition to its biocompatibility and hemo-compatibility, citric acid is also recognized as a highly germicidal chemical (Smith and Wayman, 1986; Georgopoulou et al., 1994). The reasons may be multifaceted. CA, as an organic acid, is able to flow through the cell membranes to lower the intracellular pH. Low pH within cells causes damage to enzymatic activities, protein, DNA, and extracellular membranes, thereby leading to microorganism death (Mani-López et al., 2012). Kong et al. (2001) proposed a different mechanism that organic acids such as citric acid can lower the pH and further suppress the nicotinamide adenine dinucleotide (NADH) oxidation, which results in bacteria death. Another possible reason is that citric acid alters the local pH environment and/or chelates the mental ions in the cell wall, which may prevent absorption of essential nutrients by the microorganisms due to the altered permeability of cell wall causing damage and hence cell death, especially in Gram-negative bacteria. Therefore, incorporating CA into biodegradable polymer chains is a rational approach to prevent microbial growth and can further promote many medical opportunities. Herein, we developed five different CA-derived polymers with various properties and investigated their antimicrobial behaviors in detail. These polymers, including poly(octamethylene citrate) (POC) (Yang et al., 2004), biodegradable photoluminescent polymer (BPLP) (Yang et al., 2009), crosslinked urethane-doped polyester elastomers (CUPE) (Dey et al., 2008), and poly(octamethylene maleate anhydride citrate) (POMC) (Tran et al., 2010), which are all biodegradable elastomers with tunable physical and mechanical properties. Due to their complete biodegradability, cytocompatibility, and versatility, they have been used for cardiac/bone tissue engineering, drug delivery, cell delivery, and medical imaging (Yang et al., 2004, 2006, 2009; Dey et al., 2008; Gyawali et al., 2010a,b; Tran et al., 2010, 2014; Guo et al., 2014; Xie et al., 2014). However, the potential risk of infection is always a concern. Unlike other biodegradable polymers like PLA and PLGA, CA-based polymers utilize CA not only as a monomer, but also as a potential antimicrobial agent. In this paper, we intend to evaluate the physical and intrinsic biocidal properties of CA polymers to explore their potential to prevent infections. The antimicrobial activities of these citric acid incorporated polymers against both Gram-positive *Staphylococcus aureus* (*S. aureus*) and Gram-negative *Escherichia coli* (*E. coli*) bacterium were studied to investigate the resistance of microbial-induced infection without incorporating antibiotics or other traditional biocidal agents.

## Materials and Methods

### Materials

All chemicals for polymer synthesis were purchased from Sigma-Aldrich (Milwaukee, WI, USA) and used without further purification. *E. coli* (*E. coli* 25922) and *S. aureus* (*S. aureus* 25923) were purchased from ATCC (Manassas, VA, USA). Lysogeny Broth (LB) was purchased from Sigma-Aldrich (Milwaukee, WI, USA). Bacteriostatic Hydrofera Blue wound dressing (4″ × 4″) was kindly donated by Health point (Fort Worth, TX, USA).

### Polymer synthesis

**Figure 1:**
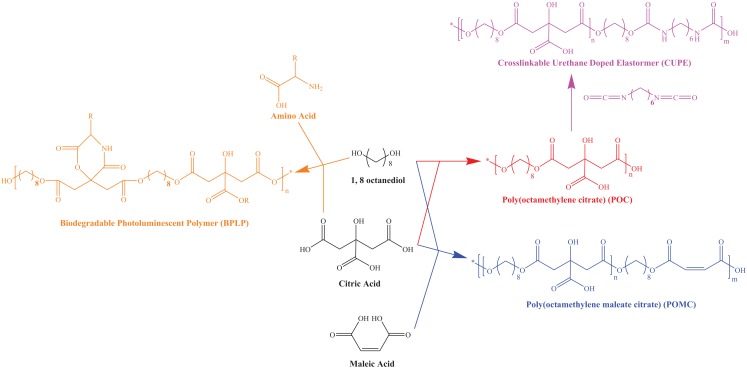
**Chemical structures and synthesis routes of citric acid based polymers including POC, BPLP, POMC, and CUPE**.

Poly(octamethylene citrate) (Yang et al., 2006), BPLP (Yang et al., 2009), CUPE (Dey et al., 2008), and POMC (Gyawali et al., 2010b; Tran et al., 2010) were synthesized according to our previous works. Generally, citric acid and other monomers were added to a 250-ml three-necked round bottom flask fitted with an inlet and an outlet adapter. The mixture was melted under a flow of nitrogen gas by stirring at 160–165°C in a silicon oil bath. The temperature of the system was subsequently lowered to 140°C under nitrogen purges and allowed to react to get different pre-polymers. The molar ratios of citric acid to other monomers are listed in Table 1. At the end of polymerization, POC, BPLP, CUPE, and POMC pre-polymers were dissolved in 1,4-dioxane and precipitated in DI water for purification. All pre-polymers were dried by lyophilization afterward. For CUPE, an additional urethane dope process was conducted as following. The POC pre-polymer was re-dissolved to make a 3% (w/w) solution in 1,4-dioxane, and then 1,6-hexamethylene diisocyanate (HDI) was added to the pre-polymer solution (1:0.9, citric acid:HDI molar ratio). Stannous octoate [Sn(Oct)_2_] was used as a catalyst to activate the reaction for one week. Further, to prepare the thermal crosslinked polymer films, all pre-polymers were heated at 80°C for 24 h in Teflon molds. Photocrosslinked POMC was prepared by using Irgacure 2959 as a photo initiator and a 365-nm ultraviolet light (UVP, Upland, CA, USA) at room temperature. All CA-based polymeric scaffolds (Porosity: 90%, pore size: 200–400 μm, thickness: 2 mm) were prepared via a salt-leaching method as we previously reported (Yang et al., 2004, 2006, 2009; Dey et al., 2008; Gyawali et al., 2010a; Tran et al., 2010). Representative schematics showing all synthesis procedures are presented in Figure 1.

**Table 1:** **Monomer ratios and crosslinking methods for CA based polymer synthesis and scaffold fabrication**.

	Molar ratio of citric acid	Type and molar ratio of diol	Type and molar ratio of the third compound	Crosslinking method
POC	1.0	1, 8-Octanediol: 1.0	None	Oven heating
BPLP	1.0	1, 8-Octanediol: 1.0	L - Cysteine: 0.2	Oven heating
CUPE	1.0	1, 8-Octanediol: 1.0	HDI: 0.9	Oven heating
POMC	0.6	1, 8-Octanediol: 1.0	Maleic acid: 0.4	Oven heating and UV exposure

### Water uptake/swelling

Crosslinked polymer scaffolds prepared as above were cut into small disks and incubated in deionized water until the equilibrium state was achieved (up to 50 h). At each time point, the surface of the swollen disks was gently blotted with filter paper to remove excess water. The samples were then weighed (*M*_wet_). The disks were again lyophilized for 3 days and weighed to determine the dry weight (*M*_dry_) Eq. 1 calculated the equilibrium-swelling ratio.

(1) $$\text{Swelling}\;\left(\%\right)=\frac{\left(M_{\text{wet}}-M_{\text{dry}}\right)}{M_{\text{dry}}}\times 100$$

### *In vitro* degradation

*In vitro* degradation studies were conducted in 10 mM PBS (pH 7.4). Six cylindrical disk specimens (7 mm in diameter; 2 mm in thickness) were cut from crosslinked polymer scaffolds. The samples were weighed, placed in a tube containing PBS (10 ml) for up to 60 days at 37°C with shaking. At the predetermined time, samples were thoroughly washed with deionized water and lyophilized for 1 week until the weight got came to equilibrium. The mass loss was calculated by comparing the initial weight (*W*_0_) with the remaining weight (*W*_t_) measured at the pre-determined time point, as shown in Eq. 2.

(2) $$\text{Mass}\;\text{remaining}\;\left(\%\right)=\frac{W_{\text{t}}}{W_{\text{0}}}\times 100$$

### Microbial culture and optical density

Gram-negative *E. coli* and Gram-positive *S. aureus* were reconstituted based on manufacturer’s instructions. Briefly, 1 ml of broth was added to rehydrate the bacterium pellet, followed by mixing a few drops of the suspension with 10 ml broth and incubating on an orbital shaker at 37°C overnight for cell expansion. First, bacteria suspension with an optical density (OD) of 0.07 at 600 nm (measured by a UV-vis spectrophotometer), which corresponded to the approximate cell density of McFarland Standard solution #1 (3 × 10^8^ CFU/ml), was prepared. Then the bacteria suspension was diluted with culture broth for 100-folds to reach the experimental concentration as previously described (Vianna et al., 2004). Dried polymer scaffold samples and Hydrofera Blue (50 mg each) were then added to 1 ml bacterial suspension and incubated with constant shaking for 0–28 h at 37°C. Scaffolds incubated with broth only were prepared as background. 2.5 μg/ml ampicillin and 29 mg/ml citric acid, which is the concentration of released citric acid from completely degraded 50 mg POC (the molar ratio of CA to 1,8-octanediol was 1:1) in 1 ml solution, were prepared separately in broth and served as positive controls in the study. All experiments were performed in aseptic conditions.

### Antimicrobial assay

Citric acid polymer films were cut into round disks with 8 mm diameters, soaked with sterile PBS for 1 h, and placed in 48 well plates. One milliliter LB broth was added to each well and the plates were vigorously shaken for 3 min. Afterward, 10 μl bacterial suspension was pipetted onto the disks and incubated for 24 h at 37°C. Hydrofera Blue and PLGA served as positive and negative controls, respectively. Colony counting was performed according to Mygrind’s method (Gottlieb et al., 2008). The antimicrobial effect of scaffolds was calculated by the percentage reduction of counted colony forming units (CFU) before and after incubation with scaffolds.

### Microbial morphology observation with SEM

Ten microlilter bacterial suspensions were seeded on top of 8 mm-diameter polymer films and incubated for 2 h at 37°C. The disks were then immediately fixed with 2.5% glutaraldehyde for 20 min and washed with PBS three times. Samples were then dehydrated in a graded ethanol series (50, 75, 95, and 100%). Finally, all samples were air-dried overnight before subjected to sputter-coating with silver and imaging by a scanning electron microscope (SEM, Hitachi S-3000N Variable Pressure, Hitachi, Pleasanton, CA, USA).

### Statistical analysis

All results were presented as mean ± SD (*n* = 6). All statistical analysis were performed with statistical significant of a 99% confidence interval (*p* < 0.01) using one-way ANOVA.

## Results

### Water uptake of CA polymer scaffolds

**Figure 2:**
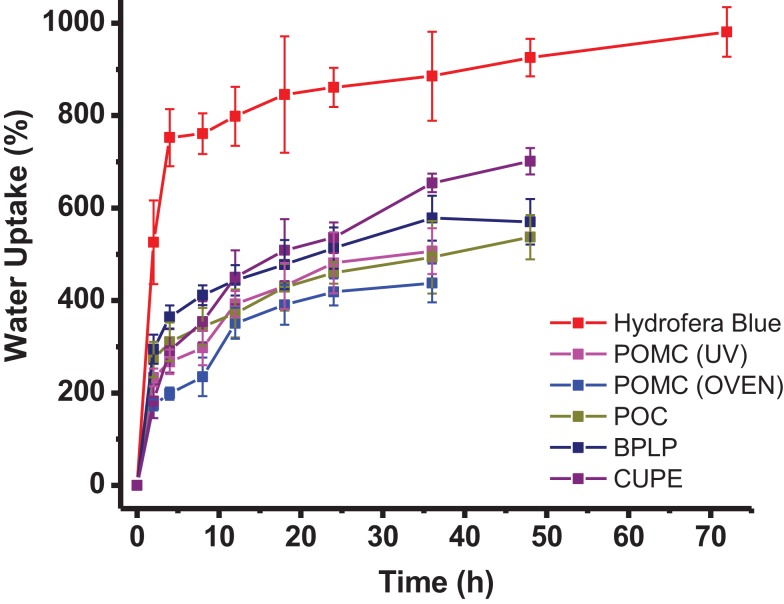
**Water uptake of crosslinked citrate-based polymers and Hydrofera Blue after different time of incubation in DI water**.

A series of CA polymers as listed in Table 1 and Figure 1 were synthesized and studied in this research. The swelling behavior of these CA-based polymers is shown in Figure 2. All polymeric scaffolds were able to uptake water, since they are crosslinked elastomers with hydrophilic carboxyl and hydroxyl functional groups. Results showed commercially available poly(vinyl alcohol) (PVA) based Hydrofera Blue had about 930 ± 52 wt% water uptake, which was significantly higher than CA polymers after 48 h incubation in DI water. The swelling ratios of CA polymers were in the range of 500–700 wt%. Specifically, CUPE exhibited the highest average uptake at 700 ± 20 wt%; while POC at 535 ± 44 wt%, and BPLP at 570 ± 46 wt%. In addition, UV crosslinked POMC scaffolds showed higher swelling ratios (~80%) than thermal crosslinked samples at 48 h, suggesting a lower crosslinking density for UV crosslinking.

### *In vitro* degradation of CA polymers

**Figure 3:**
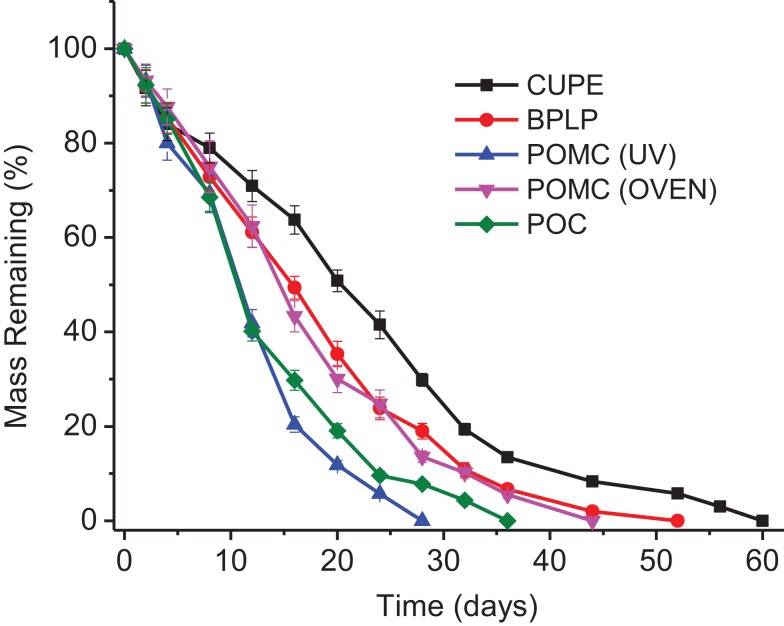
***In vitro* degradation curves of citrate-based polymers in PBS until completely degraded**.

Degradation rates of polymers vary with chemical structures, physical morphologies, and crosslinking densities. The degradation of CA-based polymers leads to a release of free CA, which could affect the intrinsic antimicrobial properties. Thus, we further explored the degradation behaviors of different CA-based polymers. The data for *in vitro* degradation of CA-based polymer disks in PBS at 37°C is presented in Figure 3, which shows that all CA-based polymers are fully degradable. Differences in degradation rates can be observed among different CA polymers, mainly due to the various crosslinking degrees, rigidity, and hydrophobicity/hydrophilicity of different polymer chains. CUPE, which is an aliphatic polyester with urethane bonds doped in the polymer network, can be degraded via hydrolysis of the ester bonds (Hafeman et al., 2011). However, CUPE degrades slower (in 60 days) than other samples because strong molecular interactions among the relatively hydrophobic urethane-doped polymer chains. POC degraded faster (36 days) than the other polymers except POMC (UV crosslinked), suggesting a faster release of free citric acid. We also noticed that thermally crosslinked POMC degraded slower in PBS than UV crosslinked POMC, which degrades the fastest among all of the tested polymers. This result is consistent with the swelling data, indicating a relatively lower crosslinking density for UV crosslinking. Additionally, CA polymers displayed increasing swelling behavior with longer incubation times due to the gradual diffusion of water and the degradation of polymers introducing porosity.

### Bacteria turbidity

**Figure 4:**
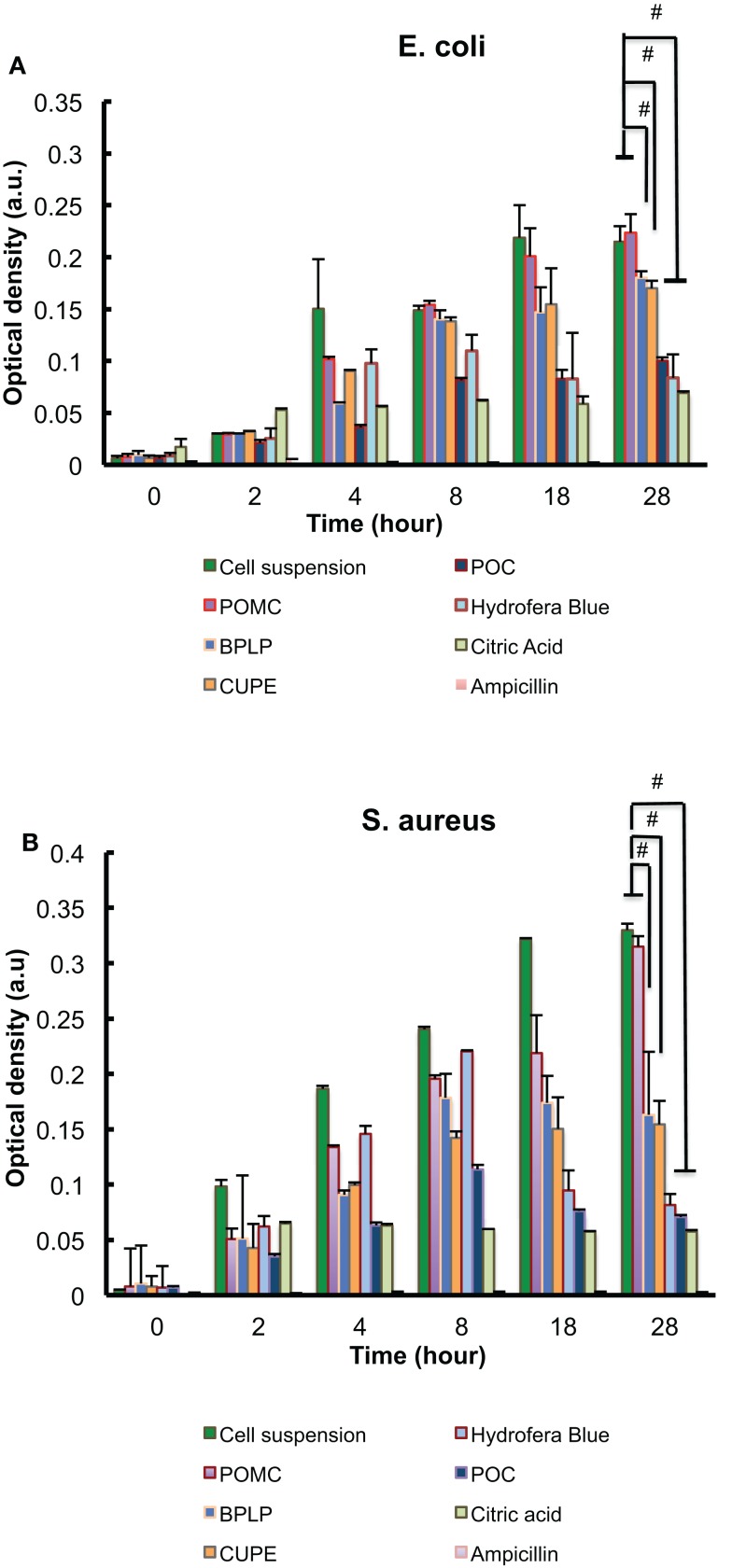
**Bacteria turbidity (optical density) studies with different citrate-based polymers (50 mg in 1 ml solution) for 28 h, in which (A) is *E. coli* and (B) is *S. aureus***. Cell suspensions without any treatment are the negative controls, while free ampicillin (2.5 μg/ml), citric acid (29 mg/ml), and commercial bacteriostatic Hydrofera Blue (50 mg in 1 ml solution) are positive controls;^#^*p* < 0.01.

The antimicrobial properties of polymers were evaluated against two bacterial strains, *E. coli* (Gram-negative) and *S. aureus* (Gram-positive). Figures 4A,B show the OD of bacteria solutions after being treated with CA-based polymers, plus negative (bacteria/cell suspension), and positive (bacteria suspension treated antibiotic ampicillin, free citric acid solution, and commercial bacteriostatic Hydrofera Blue) controls for comparison. For both bacteria strains, POC consistently demonstrated the highest suppression of bacterial proliferation among the other CA-based polymers, whereas POMC showed relatively marginal microbial inhibition where no significant difference can be observed compared to the negative control. CUPE and BPLP performed intermediate inhibition strength against microorganisms, where the differences were still significant with around 20 and 50% reduction for *E. coli* and *S. aureus*, respectively, at day 28 (*p* < 0.01). In comparison, the commercially available wound dressing, Hydrofera Blue, showed no antimicrobial property at the early time points, but gradually activated its germicidal function at the late time points due to the slow release of antimicrobial dyes, methylene blue, which is incorporated within Hydrofera Blue (Shi et al., 2010). Most importantly, Figure 4 shows that the bacteria turbidity incubated with POC scaffolds reduced 68% for *E. coli* and 83% for *S. aureus* after 28 h of incubation compared to free bacteria control indicating that POC has similar or better bacteria-killing properties compared to that of free citric acid (29 mg/ml) and commercial Hydrofera Blue samples at late time points.

### Antimicrobial assay

**Figure 5:**
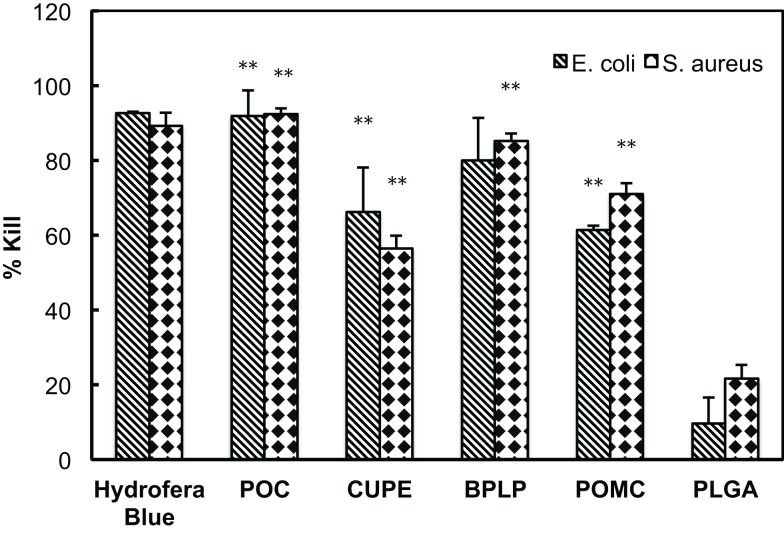
**Percentage kill of *E. coli* and *S. aureus* incubated with different CA-based polymers**. The result was calculated based on reduction of CFUs before and after bacteria suspensions incubating with CA polymers. Hydrofera Blue served as the positive control and PLGA scaffolds served as the negative control; **p < 0.01 comparing to PLGA group.

The results of antimicrobial assay are presented as the percentage of reduced CFU after incubating with CA derived polymer samples (% bacterial kill), as shown in Figure 5. Hydrofera Blue served as a positive control and a commonly used biodegradable polymer, PLGA, served as a negative control. Among all CA-based polymers, POC and BPLP had more than 80% average kill for both *E. coli* and *S. aureus*. POC showed antimicrobial effects similar to the positive control group. The % bacterial kill of POC and BPLP is significantly higher than that of PLGA (*p* < 0.01). Subsequently, CUPE and POMC showed moderate effectiveness (50–80% reduction with two types of bacterium); however, the differences in bacteria viability to that of PLGA are still significant (*p* < 0.01).

### Morphology of bacteria

Bacteria morphology on polymer films was observed by SEM to confirm CA-based polymers’ capability to inhibit bacteria growth. The representative images were taken at random positions on different polymer films with both low (800×) and high (4000×) magnification. Hydrofera Blue, which is a highly porous scaffold, served as the control. In case of Gram-negative *E. coli*, colonies mainly accumulated and aggregated at the edge of Hydrofera Blue pores. Whereas, on the surface of POC, the morphology of microbes appear relatively separated and spread with less aggregation found on center and edge areas. For Gram-positive *S. aureus*, we also explored similar behaviors for POC. *E. coli* on BPLP exhibited separated and deformed formation. However, at the edge of BPLP scaffold with *S. aureus*, we found a net-like pattern, indicating the beginning of biofilm formation (MacKintosh et al., 2006). Many cell aggregations were observed on both POMC and CUPE for both bacteria strains. Especially, the late stage of the *E. coli* biofilm appeared to be spreading on the surfaces of CUPE and POMC, suggesting their relatively weaker biocidal performance. Less cell aggregation of POC and BPLP suggested they were more effective in preventing bacteria growth.

## Discussion

Bacteria infection is a major medical complication that has hindered the usage of biodegradable polymers for medical applications. Until now, traditional biodegradable polymers such as PLA, PLGA, and poly(glycerol sebacate) (PGS), cannot prevent infections by themselves. Antibiotics, nano-silver, and other chemicals are often required to be coated or encapsulated with these biodegradable polymers in order to inhibit bacteria growth. Citric acid based polymers, BPLP, CUPE, POC, and POMC, are emerging new biodegradable materials. Due to the antimicrobial nature of citric acid, CA-based polymers could possess intrinsic antimicrobial properties, and their microbial resistant behavior and related physical properties were investigated in this study.

Citric acid-based polymers were prepared by simple polycondensation reactions, followed by thermal crosslinking. Thus, they can degrade via the hydrolysis ester bonds that can release free citric acid, which have been confirmed in our previous studies (Serrano et al., 2011; Tran et al., 2014). Adding maleic acids to polymer backbones, POMC can also be crosslinked through free radical polymerization via UV exposure. By tuning the chemical structures of these citric-based polymers, various swelling ratios (Figure 2), and degradation rates (Figure 3) were presented with potentials for different tissues engineering applications. Traditional antimicrobial elastomers and gels require incorporation of antibiotics or inorganic materials like silver nanoparticles (Thomas et al., 2007; Singh et al., 2013). Thus, high swelling ratio could lead to a burst release of these bacteria-killing agents that limits the *in vivo* anti-infection outcomes, especially for long-term (Loke et al., 2000). CA-based elastomers and hydrogels provide unique intrinsic antimicrobial properties, thus the swelling can be tailored to meet other requirements, e.g., mechanical demands, in tissue engineering without concerns of the burst release. Also, CA-based polymers can be easily manipulated physically and mechanically. Unlike the natural antimicrobial polymers, e.g., chitosan (Zhou et al., 2009), CA polymers are advantageous due to the widely tunable material properties and functionalities for different biomedical applications.

It is well known that citric acid alone is effective for preventing bacterial growth or enhancing the antimicrobial properties of other antibiotics (Ogita et al., 2009). Allende et al. (2009) compared and concluded that *E. coli* proliferation was suppressed in fresh-cut cilantro after treating the cilantro with citric acid containing solution. Fu and his group mixed citric acid as crosslinker with chitosan derivatives in cotton fabrics to create antimicrobial properties. They found that 99% of *S. aureus* and 96% of *E. coli* was killed when 14 wt% of citric acid was used in the system (Fu et al., 2011). In this work, our investigations indicated that bacteria growth was inhibited by all CA polymers in different degrees. Particularly, POC showed to be the most effective against bacteria growth and is comparable to pure CA solutions toward killing both *E. coli* and *S. aureus* in terms of the bacteria growth inhibition. In fact, POC contains a relatively higher ratio of citric acid compared to other polymers, owing to it being composed of only two monomers (1,8-octanediol and citric acid in a 1:1 molar ratio) involved in synthesis. POC also degraded faster than other CA polymers except that POMC is UV crosslinked (Figure 3), leading to a quicker release of free citric acid, which is the active antimicrobial compound. Interestingly, after 28 days of incubation and degradation, POC exhibited similar bacteria inhibition for both *E. coli* and *S. aureus* as free citric acid with the concentration of completely degraded POC. CA, one of the organic carboxylic acids (Allende et al., 2009), contributes to local pH reduction, which may depress the internal pH of bacteria and/or alter the permeability of microbial membrane by disrupting their substrate transport (Kong et al., 2001; Bischof Vukusic et al., 2011; Mani-López et al., 2012). Thus, the unreacted carboxyl groups on CA-based polymers and degradation-released citric acid could be responsible for antimicrobial behaviors.

**Figure 6:**
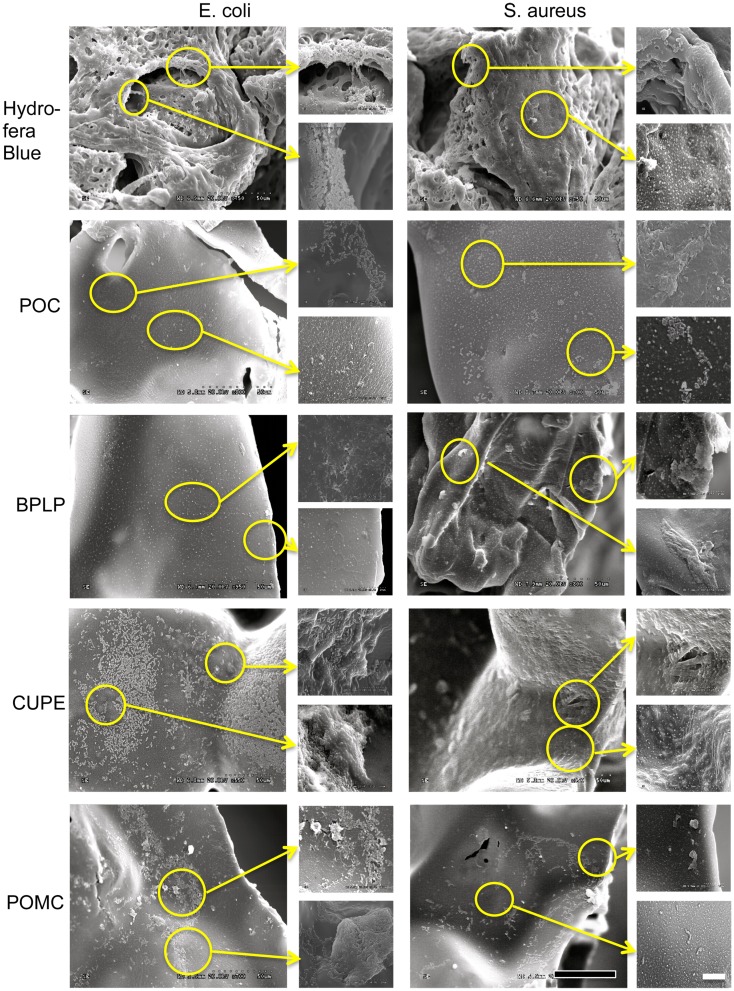
**SEM images of microbes (*E. coli* and *S. aureus*) on the surface of different citrate-based polymers and Hydrofera Blue. (Black scale bar for 800× magnification = 50 μm; White scale bar for enlarged 4000× magnification = 10 μm)**.

Based on the *in vitro* microbial morphology, four typical steps of bacterial proliferation can be observed: (1) cells adhere on material surface; (2) cells accumulate and aggregate in multiple layers; (3) biofilm formation and maturation; (4) cells detach from the biofilm to a planktonic state for a new cycle of bacterial proliferation (Mack et al., 2004). Zhou et al. (2011) described that bacteria surfaces became wrinkled and withered after incubating with their antimicrobial hydrogel coating. In addition, Otto et al. (2010) observed *E. coli* lyse, representing cell death, when samples were treated with mineral leachates. Our SEM images revealed various morphologies of bacterial activities on CA-based polymers. As demonstrated in Figure 6, different CA based materials showed different levels of bacterial inhibition. For example, *E. coli* and *S. aureus* tend to distribute evenly on POC and BPLP. However, cell aggregations were formed on BPLP (*S. aureus* only), CUPE, and POMC in different degrees. Biofilms, as a later stage of bacteria growth, were found on CUPE and POMC. However, some of them showed similar bacteria morphology as commercial bacteriostatic Hydrofera Blue. Essentially, bacteria aggregated much less on POC than other CA polymer and Hydrofera Blue. This result is consistent with previous *in vitro* assay results. Therefore, these citric acid containing polymers surely disturbed normal microbe survival/growth patterns in different degrees.

## Conclusion

In this study, four different biodegradable citric-based materials have been synthesized and characterized for their antimicrobial related properties. These materials can be tuned to have various physical properties including water uptake and degradation rate according to their potential applications. Among the investigated citric-acid based polymers (BPLP, CUPE, POC, and POMC), POC has the highest antimicrobial properties by taking advantage of its higher citric acid ratio and faster degradation rate, while other polymers exhibited moderate bacteria inhibition. This is the first report to systematically evaluate the antimicrobial properties of CA-based polymers without the induction of additional antibiotics, peptides, or inorganic materials, which should reaffirm the growing interests in using CA-based polymers for a number of biomedical applications, especially where antimicrobial properties are desired.

## Conflict of Interest Statement

The authors declare that the research was conducted in the absence of any commercial or financial relationships that could be construed as a potential conflict of interest.
